# How is city living associated with psychosis? Findings from a novel data linkage of 612,988 people from an urban and ethnically diverse area

**DOI:** 10.23889/ijpds.v8i2.2264

**Published:** 2023-09-14

**Authors:** Rosanna Hildersley, Jayati Das-Munshi, Peter Schofield, Lukasz Cybulski, Milena Wuerth

**Affiliations:** 1 King's College London, London, United Kingdom

## Objectives

UK cities show higher incidence of psychotic disorders, but the reasons remain unclear. This case-control study uses data from one of the first and largest person-level data linkages between mental health records and the UK census to explore associations previously only assessed using ecological or smaller studies in England.

## Methods

The SocioEconomic Predictors of Mental Disorders (SEP-MD) project dataset comprises of data extracted from electronic health records (EHR) from the South London and Maudsley NHS Foundation trust (SLaM). These EHRs were linked to the 2011 UK census as a collaboration between SLaM, the ONS and King’s College London.

Cases with clinical diagnoses of non-affective (schizophrenia-spectrum) and affective psychoses (bipolar disorder, depression with psychosis) were identified. Population controls were sampled from the locality.

Logistic regression models were used to calculate weighted adjusted (age and sex) odds ratios (waOR) to assess associations. Robust standard errors were used to account for clustering.

## Results

16,863 linked cases with psychosis (affective n=5,694; non-affective n=11,169) were identified alongside 596,125 population controls.

Cases with psychosis were more likely to live in areas with the highest population density (waOR 1.17 (1.05, 1.30)) when comparing the lowest quintile to the highest. Non-affective disorders showed the highest association with population density.

Being born within the UK was associated with a higher risk of psychosis, and migrants living in the country for longer were at a significantly higher risk than those living in the UK for less time. Socioeconomic predictors, including education, occupation and tenure, were all associated with higher psychosis risk. Racialised minorities were at higher risk of specifically non-affective psychoses. Indicators of isolation (marital status and living alone) were highly associated with psychosis risk.

## Conclusion

Our findings regarding urbanicity, ethnicity, migration socioeconomic position and social circumstances both confirm and provide further depth to previously identified associations. Novel findings relating to migration and interactions with ethnicity will require further investigation.

These insights will provide valuable information for future public health and policy development.

